# A Comparative Study of Topical 2% Diltiazem Versus Lateral Internal Sphincterotomy in the Treatment of Fissure-in-Ano

**DOI:** 10.7759/cureus.98615

**Published:** 2025-12-07

**Authors:** Simhadri Siripurapu, Rajani Devi Gosala, Sri Satya Sudha Tula, Vijaya Petta, Kusa R Pyla

**Affiliations:** 1 General Surgery, Rangaraya Medical College, Kakinada, IND; 2 Plastic Surgery, Rangaraya Medical College, Kakinada, IND; 3 Pathology, Rangaraya Medical College, Kakinada, IND

**Keywords:** 2% diltiazem cream, chronic anal fissure, effectiveness of healing, lateral anal sphincterotomy, recurrence

## Abstract

Introduction: Chronic anal fissure is a common clinical entity seen in surgical practice. Lateral internal sphincterotomy is considered the gold standard for treating chronic fissures. But recent developments in understanding the pathophysiology of anal fissures have helped to develop better conservative treatment options. In this study, we compare the healing, symptomatic relief, and side effects of topical 2% Diltiazem gel versus lateral internal sphincterotomy in the treatment of chronic anal fissures.

Material and methods: In this prospective, randomised comparative study, 100 patients with chronic anal fissure were randomly divided into group A (Diltiazem gel) and group B (lateral internal sphincterotomy). Each group has 50 patients. Patients were followed up at the first, second, and third weeks and first, second, and third months, and symptomatic relief, healing, and side effects were noted for each group.

Results: More than 90% of the subjects in group A had a pain scale ≤ 3. Among group B, two subjects (4%) had pain rated as 4 after two weeks of treatment. The rate of healing in patients belonging to groups A and B is 82% and 68%, respectively, at the end of three months. In group A, one patient and, in group B, 15 patients showed flatus incontinence at six months. In group A, six (12%) patients and, in group B, one (2%) patient had recurrence.

Conclusion: Study concludes that lateral internal sphincterotomy remains the gold standard treatment for chronic anal fissure. Diltiazem therapy is a good alternative for patients who refused surgery or prefer medical line of treatment and can be used as a first choice in management of chronic fissures.

## Introduction

An anal fissure is a common and painful condition that affects the anal canal. It is characterized by a tear or crack in the mucosa that exposes the underlying muscle. The main symptom is severe pain during and after defecation, often accompanied by bleeding. An anal fissure can be acute or chronic, depending on the duration and healing of the lesion. A chronic anal fissure is defined as a fissure that persists for more than six weeks and is associated with hypertonia and spasm of the internal anal sphincter [1].

The treatment of chronic anal fissures aims to relieve pain, promote healing, and prevent recurrence. The standard surgical treatment is lateral internal sphincterotomy, which involves cutting a part of the internal anal sphincter to reduce the resting pressure and improve blood flow to the fissure. However, this procedure has some drawbacks, such as the risk of incontinence, infection, and recurrence. Therefore, alternative treatments have been sought to avoid surgery and its complications.

One of the most promising alternatives is the use of topical 2% Diltiazem, ability to decrease calcium influx into the smooth muscle cells of internal anal sphincter that causes relaxation of the smooth muscle, and chemical sphincterotomy. 2% Diltiazem ointment is applied to the perianal area, usually twice a day, for several weeks. Several studies have shown that 2% Diltiazem can heal chronic anal fissures in 50% to 70% of cases. However, 2% Diltiazem has some limitations, such as poor compliance, low healing rate, and high recurrence rate.

This study aims to compare the efficacy and safety of topical 2% Diltiazem and lateral internal sphincterotomy in the treatment of anal fissures. The term "Fissure-in-Ano" refers to a mucosal split that runs from the anal margin to the dentate line and was initially used by Lockhart-Mummery in 1934 [2]. Among adults, it is the most common cause of severe anorectal discomfort, with an estimated 0.11% annual incidence. An ulcer or little tear in the anal canal lining is the hallmark of a frequent ailment called a fissure-in-ano. It normally appears close to the anus and can be extremely painful and uncomfortable. The most common causes are as follows: chronic straining during bowel movements can lead to the development of fissures; passing hard or large stools can traumatize the delicate anal tissue; injury due to excessive wiping, insertion of foreign objects, or forceful dilation during childbirth can cause tissue damage [1]; and persistent muscle spasm around the anus can impair blood flow and delay healing.

A rip in the anal mucosa is an example of an early trauma that frequently precedes fissure-in-ano. Things like hard stools, constipation, or undue straining during bowel movements might cause this stress. Following the first injury, the internal anal sphincter (IAS) frequently experiences hypertonicity. Increased muscle tone in the IAS exacerbates pain and the fissure. Local ischemia, or decreased blood flow, is caused by a compromised blood supply to the affected area. Ischemia prolongs the pain-spasm cycle and hinders tissue repair. The fissure initiates a never-ending cycle in which pain exacerbates hypertonicity and ischemia by inducing muscle spasm. This erratic behaviour keeps the symptoms ongoing.

Males and females both have fissures at the same frequency. With a mean age of onset of 39.9 years, fissures are most frequently observed in middle-aged and younger patients. Elderly people and youngsters can also develop fissures. The posterior midline is the most prevalent spot for both males and females; over 75% of cases occur here [1]. The anterior site accounts for around 25% of fissures, and these are more prevalent in women. Atypical fissures are those that make up less than 1% of all fissures and are situated apart from the midline location. Atypical fissure location (other than usual midline position) was found in diseases like Crohn's illness, ulcerative colitis, anal cancer, tuberculosis, HIV, syphilis, herpes, and leukaemia [1].

The posterior midline is the most typical location for primary anal fissures, and there are various explanations explaining this phenomena [3]. The external sphincter's elliptical posterior configuration, which provides the anal canal with less support, is one potential reason. The relative ischemia of the anal canal's posterior commissure is another potential reason. Less blood flow has been observed in postmortem angiographic studies to the posterior commissure of the anal canal, which could also account for the higher incidence of posterior midline fissure. Additionally, there might be a contusion of the blood vessels that go through the posterior midline's internal sphincter muscle vertically, which would compromise the blood supply and raise anal tone. Blood flow to the posterior midline is lower than to other areas of the anal canal, as demonstrated by Doppler laser flowmetry combined with anal manometry. Additionally, there is an inverse association between blood flow to the posterior commissure and increased sphincter tone [4].

## Materials and methods

The current study was done in the General Surgery Department, Rangaraya Medical College and General Hospital, Kakinada, in patients with a fissure-in-ano. The study period is 12 months from July 2024 to August 2025. A prospective, randomised, interventional, open-label study was conducted to compare the efficacy of lateral sphincterotomy with topical 2% Diltiazem in the treatment of anal fissures. The data were collected from 100 patients with fissure-in-ano attending the Department of General Surgery, who were included in the study. This study was approved by Rangaraya Medical College, Kakinada, India (Approval No: REG.NO.IEC/RMC/2024/1310, dated: May 6, 2024).

Calculation of the sample size

According to the study done by Khan et al., the prevalence of anal fissure was 15% [5].

N=Z2PQ/E2

Where N is the sample size (N=100), P is the prevalence, Q=1-P, E is the margin of error (7%), and Z is the constant number for 95% confidence levels. Hence, a total of 100 patients who willingly gave their written informed consent were included for assessment of outcome.

Inclusion criteria

All patients presenting with fissure-in-ano and meeting the following criteria were included: age group >18 years; both males and females; and anal fissures associated with features of chronicity like sentinel pile, hypertrophied papillae, or exposure of horizontal fibres of internal sphincter.

Exclusion criteria

Patients with the following criteria are excluded: other causes of bleeding pulse rate (PR) like haemorrhoids and fistula-in-ano, inflammatory bowel disease, and rectal malignancy; previous surgeries in anal canal; patients below the age <18 years; acute fissure-in-ano; patients on certain drugs, including aminoglycosides, baclofen, dantrolene, diazepam, anticoagulants, and other vasodilators; and persons who have known hypersensitivity reaction to 2% Diltiazem.

Methodology

A total of 100 patients included in this study were subjected to preoperative assessment including the following: complete history taking: pain, bleeding, constipation, pruritus, and discharge; clinical examination (general and local): anorectal examination, to detect the site of the fissure confirming its nature and presence of associated anal pathology; and laboratory investigations: it included haemoglobin%, total count (TC), differential count (DC), platelet count (PLTC), bleeding time (BT), clotting time (CT), international normalised ratio (INR), urine albumin, sugar, renal function test (RFT), liver function test (LFT), fasting blood sugar (FBS), chest X ray (CXR), and ECG. All patients were categorized into mild, moderate, and severe according to the perception of pain. A total of 100 patients were randomly allocated into two groups of 50 patients each: group A (2% Diltiazem); and group B (lateral internal sphincterotomy (LIS) group).

A total of 50 patients in group B were surgically treated with open internal sphincterotomy (LIS); the procedure was performed under general anaesthesia without the use of muscle relaxants. Four hours after the procedure, the patient began a liquid diet, and eight hours later, the anal dressing was taken off. Before defecating, a local anaesthetic cream was used. On the next postoperative day, the patients in this group were given instructions on a high residue food, painkillers, and warm sitz baths.

Application guidelines for the 2% Diltiazem were provided. For eight weeks, all patients in group A were instructed to apply 2% Diltiazem ointment on the external anal margin three times a day. Patients whose fissures had not healed at the time of the initial review were asked to stay on 2% Diltiazem for an additional four weeks before being reviewed once more.

Follow-up criteria

Patients were followed up in Out Patient Department for relief of pain, fissure healing, post-treatment bleeding, infection, and incontinence, as well as recurrence and satisfaction. Follow-up was carried out at first week, second week, first month, second month, and third month after treatment. During the follow-up, the success of treatment and improvement of the previous symptoms were evaluated. Healing and recurrence rates were determined, the healing defined as complete re-epithelialization of the fissure site as documented by the physician. Recurrence was defined as fissure identified after documentation of complete healing. Incontinence was categorized as soiling of underclothing or lack of flatus control.

## Results

Among the 100 subjects that were included in the study, 50 subjects with anal fissure who received topical 2% Diltiazem were categorised as group A. Fifty subjects with anal fissure had undergone lateral sphincterotomy and are categorised as group B (Table 1).

**Table 1:** Subjects Categorisation.

Treatment	Frequency	Percent
Topical 2% Diltiazem (group A)	50	50.00%
Lateral sphincterotomy (group B)	50	50.00%
Total	100	100.00%

Age and gender

Out of the 100 patients, there were 53 females and 47 males. The male-to-female ratio in the present study is 0.88 implicating female predominance of the disease presentation. The commonest age group presented with anal fissure in the study was 41 to 50 years followed by 31 to 40 years in both the genders. The number of females affected with anal fissure is higher when compared to males and is statistically significant. Mean age of subjects in group A is 39.14 ± 7.5 years. Mean age of subjects in group B is 38.6 ± 11.0 years. The minimum age of the subjects in group A is 20 years. The minimum age of the subjects in group B is 20 years. The maximum age of the subjects in group A is 54 years. The maximum age of the subjects in group B is 54 years. No significant statistical difference in the mean age of the study subjects is observed (p=0.77) (Table 2).

**Table 2:** Age and Gender.

	Gender		
Age category (years)	Females	Males	Total
20 to 30	7	17	24
31 to 40	19	8	27
41 to 50	26	19	45
51 to 60	1	3	4
Total	53	47	100

Position of fissure

The anal fissure is posteriorly present anatomically in 64% of study group. This is the commonest presentation. Thirty-six percent of study population had it anteriorly. Among 36 patients with anteriorly located fissure-in-ano, 23 (63.8%) patients were treated with topical 2% Diltiazem. In patients with anal fissures which were posteriorly present anatomically, 37 (57%) patients were treated with lateral sphincterotomy. There is significant statistical difference in patients with anal fissure who presented either anteriorly or posteriorly (Chi-square value=3.5, p value=0.06) (Table 3).

**Table 3:** Position of Anal Fissure.

	Group		
Position of fissure	A	B	Total
Anterior	23	13	36
Posterior	27	37	64
Total	50	50	100

Gender and position of fissure

Among 36% of the patients, in whom the fissure was present anteriorly, 21 (58%) were females and 15 (42%) were males. Among 64% patients in whom the anal fissure was present posteriorly, males and females were equally distributed (50% each). Although differences exist in the frequency of males and females with respect to position of anal fissure, no significant association is seen (Chi-square value=0.35, p value=0.55) (Table 4).

**Table 4:** Position of the Anal Fissure and Gender.

	Gender		
Position of fissure	Female	Male	Total
Anterior	21	15	36
Posterior	32	32	64
Total	53	47	100

Pain (Visual Analog Scale) after 24 hours of treatment

Visual Analog Scale (VAS) score is 4 in 21% (8) of Diltiazem study subjects, where 61% (13) of them were treated with lateral sphincterotomy [6]. Among 47 subjects with VAS score of 3, 55% (26) have been treated with lateral sphincterotomy. Among 32 subjects with VAS score of 2, majority (65%) had 2% Diltiazem as the treatment. Majority of patients who received topical 2% Diltiazem for anal fissure had pain at low level (VAS: 2). Majority of patients who received lateral sphincterotomy for anal fissure had pain at higher level (VAS: 4, 3). But this difference in pain level among the two treatment groups is statistically non-significant (Table 5).

**Table 5:** VAS: 24 Hours After Treatment. VAS: Visual Analog Scale.

	Treatment		
Pain after 24 hours of treatment (VAS score)	2% Diltiazem	Sphincterotomy	Total
2	21	11	32
3	21	26	47
4	8	13	21
Total	50	50	100

Pain at 2 weeks after treatment

Among 50 subjects treated with topical 2% Diltiazem, three (6%) subjects had pain rated as 4, 20 subjects (40%) had pain rated as 3, 17 subjects (34%) had pain rated as 2, and 10 subjects (20%) had pain rated as 1 after two weeks of treatment. More than 90% subjects treated with topical 2% Diltiazem had a pain score of ≤ 3. Among 50 subjects managed with lateral sphincterotomy, two subjects (4%) had pain rated as 4, 26 subjects (52%) had pain rated as 3, 16 subjects (32%) had pain rated as 2, and six subjects (12%) had pain rated as 1 after two weeks of treatment. Only half of the subjects treated with lateral sphincterotomy were having pain rated as 2 even after two weeks of treatment. There is no significant difference in pain perception in both the groups at two weeks after treatment (Chi-square value=3.01, p value=0.56) (Table 6).

**Table 6:** VAS: Two Weeks After Treatment. VAS: Visual Analog Scale.

	Treatment		
Pain at 2 weeks (VAS score)	Diltiazem	Sphincterotomy	Total
1	10	6	16
2	17	16	33
3	20	26	46
4	3	2	5
Total	50	50	100

Patient satisfaction

Among 50 subjects managed with topical 2% Diltiazem, 42 (84%) of them were satisfied; only eight (16%) of them were not satisfied. Among 50 subjects managed with lateral sphincterotomy, 26 (52%) of them were satisfied; 24 (48%) of them were not satisfied. A significant number of subjects were satisfied with the application of topical 2% Diltiazem when compared to lateral sphincterotomy in this study (p=0.0013) (Table 7).

**Table 7:** Patient Satisfaction.

	Treatment		
Patient satisfaction at first month	Diltiazem	Lateral sphincterotomy	Total
No	8	24	32
Yes	42	26	68
Total	50	50	100

Rates of healing

Among 50 subjects who were treated with topical 2% Diltiazem, 41 (82%) of them were healed and only nine (18%) of them were not healed. Among 50 subjects treated with lateral sphincterotomy, 34 (68%) of them were healed. Among 50 subjects treated with lateral sphincterotomy, 16 (32%) of them were not healed. Rate of healing in patients belonging to groups A and B is 82% and 68%, respectively. Hence, the rate of healing was more in patients who were treated with topical 2% Diltiazem when compared to lateral sphincterotomy but is statistically not significant (p=0.165) (Table 8).

**Table 8:** Rate of Healing.

	Treatment		
Healing	Diltiazem	Lateral sphincterotomy	Total
No	9	16	25
Row%	36.00%	64.00%	100.00%
Col%	18.00%	32.00%	25.00%
Yes	41	34	75
Row%	54.67%	45.33%	100.00%
Col%	82.00%	68.00%	75.00%
Total	50	50	100
Row%	50.00%	50.00%	100.00%
Col%	100.00%	100.00%	100.00%

Incontinence

One patient (2%), out of 50 patients treated with topical 2% Diltiazem, remained incontinent even after six months of treatment. Among 50 subjects treated with lateral sphincterotomy, 15 (30%) of them had incontinence. Among 16 patients who had rectal incontinence, majority (15 (93%) patients) were belonging to group B. Hence, the rate of rectal incontinence was more in patients who received lateral sphincterotomy which is statistically significant (p=0.0003) (Table 9).

**Table 9:** Rate of Incontinence.

	Treatment		
Incontinence at 6 months	Diltiazem	Lateral sphincterotomy	Total
No	49	35	84
Row%	58.33%	41.67%	100.00%
Col%	98.00%	70.00%	84.00%
Yes	1	15	16
Row%	6.25%	93.75%	100.00%
Col%	2.00%	30.00%	16.00%
Total	50	50	100
Row%	50.00%	50.00%	100.00%
Col%	100.00%	100.00%	100.00%

Rate of recurrence

Among 50 subjects treated with topical 2% Diltiazem, six (12%) patients had recurrence of anal fissure. Among 50 subjects treated with lateral sphincterotomy, one (2%) patient with anal fissure had recurrence. Among seven patients who had recurrence of anal fissure, majority (six (85%) patients) were belonging to group A. Hence, the rate of recurrence was less in patients who received lateral sphincterotomy which is statistically significant (p=0.0003) (Table 10).

**Table 10:** Recurrence Rate.

	Treatment		
Recurrence at 6 months	2% Diltiazem	Lateral sphincterotomy	Total
No	44	49	93
Row%	47.31%	52.69%	100.00%
Col%	88.00%	98.00%	93.00%
Yes	6	1	7
Row%	85.71%	14.29%	100.00%
Col%	12.00%	2.00%	7.00%
Total	50	50	100
Row%	50.00%	50.00%	100.00%
Col%	100.00%	100.00%	100.00%

Duration of hospital stay

Among 50 patients with anal fissure who were treated with topical 2% Diltiazem, duration of hospital stay was increased in five (10%) patients. Forty-one (82%) of the 50 patients with anal fissure who underwent lateral sphincterotomy had longer hospital stays. Of the 54 patients with anal fissure who were in the hospital for a normal amount of time, most were in group A. Hence, more number of patients belonging to group B had prolonged duration of hospital stay and is statistically highly significant (p<0.001) (Table 11).

**Table 11:** Hospital Stay.

	Treatment		
Duration of stay	Diltiazem	Lateral sphincterotomy	Total
Increased	5	41	46
Row%	10.87%	89.13%	100.00%
Col%	10.00%	82.00%	46.00%
Normal	45	9	54
Row%	83.33%	16.67%	100.00%
Col%	90.00%	18.00%	54.00%
Total	50	50	100
Row%	50.00%	50.00%	100.00%
Col%	100.00%	100.00%	100.00%

## Discussion

Anal fissure is a prevalent issue in healthcare that plagues both patients and doctors. The majority heal by conservative treatment, but a considerable percentage become chronic. A total of 100 patients with chronic anal fissure attending the Out Patient Department (OPD) of the General Surgery Department in Rangaraya Medical College, Kakinada, were studied. The effect of topical 2% Diltiazem and lateral sphincterotomy in the treatment of anal fissure is compared.

Comparison between categorical findings and associations was done using the Chi-square test. A non-parametric test is used when the data is qualitative. Mean and SD were expressed for numerical values like age. Most of the variables were also expressed using frequency and percentages.

Demographics

Mean age of subjects in group A is 39.14 ± 7.5 years. Mean age of subjects in group B is 38.6 ± 11.0 years. No significant statistical difference in the mean age of the study subjects is observed (p=0.77). Out of the 100 patients, there were 53 females and 47 males.

Pain after intervention

Pain is assessed with the help of Visual Analogue Scale in the present study. It is assessed 24 hours after the intervention (2% Diltiazem and lateral sphincterotomy) and two weeks after the intervention. Visual Analogue Scale (VAS) score assessed 24 hours after intervention: VAS score of 4 is observed in 21% (8) of Diltiazem group subjects and 61% (13) lateral sphincterotomy group. Among 47 subjects with VAS score of 3, 55% (26) have been treated with lateral sphincterotomy. Among 32 subjects with a VAS score of 2, the majority (65%) had topical 2% Diltiazem as the treatment. The majority of patients who received topical Diltiazem for anal fissure had pain at a low level (VAS: 2). The majority of patients who received lateral sphincterotomy for anal fissure had pain at a higher level (VAS: 4, 3) [7]. But this difference in pain level among the two treatment groups is statistically non-significant assessed 24 hours after the intervention in the present study.

Among 50 subjects treated with 2% Diltiazem, three (6%) subjects had pain rated as 4 after two weeks of treatment, 20 subjects (40%) had pain rated as 3 after two weeks of treatment, 17 subjects (34%) had pain rated as 2 after two weeks of treatment, and 10 subjects (20%) had pain rated as 1 after two weeks of treatment. More than 90% subjects treated with topical 2% Diltiazem were having pain scale ≤ 3. Among 50 subjects managed with lateral sphincterotomy, two subjects (4%) had pain rated as 4 after two weeks of treatment, 26 subjects (52%) had pain rated as 3 after two weeks of treatment, 16 subjects (32%) had pain rated as 2 after two weeks of treatment, and six subjects (12%) had pain rated as 1 after two weeks of treatment. Only half of the subjects treated with lateral sphincterotomy were having pain rated as 2 even after two weeks of treatment. No significant difference existed in pain perception in both the groups at two weeks after treatment (Chi-square value=3.01, p value=0.56).

Effectiveness of the interventions

Healing

Rate of healing is assessed at the end of one week post-intervention in the present study. In patients treated with 2% Diltiazem for anal fissures, the healing rate is 83%; in patients treated with lateral sphincterotomy, the rate is 68%. Higher rate of healing is observed in group treated with 2% Diltiazem but is statistically non-significant. This implicates that both the interventions had similar healing rates [8]. Suvarna et al. reported a healing rate of 69.23% with 2% topical Diltiazem gel and healing rate of 95.87% with lateral internal sphincterotomy [9].

Incontinence

Rectal incontinence is also assessed at the end of one week post-intervention in the present study. Among patients who were treated with topical 2% Diltiazem, rate of rectal incontinence is observed to be 2%, while it is 30% in those who were treated with lateral sphincterotomy. Higher rate of incontinence is observed in group treated with lateral sphincterotomy and is statistically significant. This implicates that topical 2% Diltiazem is better compared to lateral sphincterotomy with respect to rectal incontinence [10].

Recurrences

Six (85%) of the seven patients who experienced an anal fissure recurrence belonged to group A and received topical 2% Diltiazem treatment in the current investigation.

Hospitalisation

A duration of three days in-hospitalisation is mandated for every patient with anal fissure included in the study. More than three days of in-hospitalisation is considered as increased stay [11]. Out of 50 patients who were treated with topical 2% Diltiazem, duration of hospital stay was increased in five (10%) patients, while those who were treated with lateral sphincterotomy, duration of hospital stay was increased in 41 (82%) patients [12]. Hence, more number of patients belonging to group B had prolonged duration of hospital stay and is statistically highly significant (p<0.001).

Safety of the intervention

Head ache is the only adverse event encountered among patients treated with chemical sphincterotomy (five patients out of 50), while hematoma seen in four patients out of 50 is observed among patients with anal fissure intervened by lateral sphincterotomy in the present study [12].

Patient satisfaction

Among 50 subjects managed with lateral sphincterotomy, 26 (52%) of them were satisfied and 24 (48%) of them were not satisfied. A significant (p=0.0013) number of subjects were satisfied with topical 2% Diltiazem when compared to lateral sphincterotomy in the present study.

## Conclusions

Anal fissure is a common problem vexing patients and treating physicians. The majority heal by conservative interventions, but a significant proportion turn chronic and can have a negative impact on quality of life. While lateral anal sphincterotomy is the gold standard for the management of chronic fissure-in-ano, a number of non-surgical techniques have been developed without subjecting the patients to surgery. Topical 2% Diltiazem is one of the non-surgical techniques that is safe and cost-effective. It is easy to apply, well-tolerated, and has minimal side effects. Although higher rates of healing can be achieved without the risk of incontinence, the slower rate of symptomatic relief and longer duration of treatment will result in lower compliance and higher recurrence.

Lateral anal sphincterotomy is superior to non-surgical therapies, offering early symptomatic relief, rapid healing, better compliance, and a permanent cure of fissure with lower recurrence rates. Therefore, topical 2% Diltiazem/medical sphincterotomy can be considered as an initial choice of treatment for fissure-in-ano. It can be considered as the first option in patients with high chances of incontinence before surgery, who are not willing to undergo surgery and not fit for surgery. Lateral anal sphincterotomy can be reserved for patients with severe, disabling pain, not responding to medical therapy, and in recurrent and persistent anal fissures, achieving quick curative results.
